# Examining Medicare Reimbursement Differences for Cochlear Implant Surgery

**DOI:** 10.7759/cureus.99630

**Published:** 2025-12-19

**Authors:** Layla Ali, Samuel Salib, Iyawnna Hazzard, Julia Howard, Cameron Ostroot, Ahmed Salem, Richard S Isaacs

**Affiliations:** 1 College of Medicine, California Northstate University College of Medicine, Elk Grove, USA; 2 Otolaryngology - Head and Neck Surgery, California Northstate University College of Medicine, Sacramento, USA; 3 Research, California Northstate University College of Medicine, Elk Grove, USA; 4 Otolaryngology, California Northstate University College of Medicine, Elk Grove, USA

**Keywords:** cochlear implants, medicare data, medicare reimbursement trends, neuro-otology, sensorineural hearing loss

## Abstract

Objectives

This study aims to investigate discrepancies in Medicare reimbursements for cochlear implant surgery between the hospital setting and ambulatory surgery centers (ASCs), which provide a convenient alternative to hospital-based outpatient procedures by offering same-day surgical healthcare.

Study design

A retrospective analysis of Medicare claims data from 2019 to 2021 was conducted, including 358 otolaryngology and ASC providers. The study evaluated average Medicare reimbursement for cochlear implantation procedures performed in ASCs using Current Procedural Terminology (CPT) code 69930 and compared these with reimbursements for the same procedure in outpatient hospital settings using Ambulatory Payment Classification (APC) code 5166.

Methods

The primary outcome variable was the average Medicare reimbursement amount for each provider location (ASCs vs. outpatient hospital setting) for each code associated with cochlear implant surgery. Unpaired two-tailed t-tests (p <0.05) were used to compare average reimbursement amounts between the two groups. Additionally, box-and-whisker plots were utilized to illustrate the distribution of reimbursement amounts between each provider group.

Results

Outpatient hospital settings received significantly greater reimbursements for cochlear implant surgery between 2019 and 2021 (for 2019, mean = 29595.58, SD = 3934.898; for 2020, mean = 31464.87, SD = 4177.054; for 2021, mean = 32549.87, SD = 4402.242) compared with ASCs(for 2019, mean = 23202.01, SD = 2242.805, p < 0.0001; for 2020, mean = 24986.88, p < 0.001, SD = 3367.238; for 2021, mean = 26028.86, SD = 3184.769, p < 0.0001).

Conclusions

Identifying discrepancies in Medicare reimbursement for cochlear implant surgery between ASC surgeons and outpatient settings is crucial for understanding treatment selection. This study investigates current reimbursement practices and highlights areas for potential improvement within the Medicare system. Further research may explore reasons for reimbursement variations and their impact on patients needing cochlear implants.

## Introduction

Hearing loss affects approximately 15% of adults in the United States, yet only around 16% of adults, ages 20-69, who would benefit from assisted hearing devices actually undergo treatment [1]. Long-term, untreated hearing loss in elderly patients specifically has been linked with more depressive symptoms, lower self-efficacy, increased feelings of loneliness, and a smaller social network [2]. Cochlear implantation (CI) procedures are an option patients can take for correcting their hearing loss. CI procedures involve the surgical implantation of a device that can receive surrounding sounds and transform these sound signals directly to the cochlear nerve to be processed and heard by the patient [2]. However, although there is a clear benefit in completing CI for those who qualify, prior studies have shown that people with a lower socioeconomic background are less likely to complete CI, even though they are more likely to have hearing loss and qualify for the procedure [3]. This highlights the imbalanced nature of receiving proper intervention for hearing loss based on socioeconomic background.

Moreover, the majority of elderly people with lower socioeconomic status (SES) in the United States rely on Medicare to fund their medical expenses; only 5.3% of adults over 65 years old in the United States are covered solely by private insurance, and 44.8% are covered by Medicare alone as of 2022 [4].

High out-of-pocket costs are a possible reason why such patients do not undergo the CI procedure. Medicare is a federal health insurance program that provides medical payments for adults over 65 years of age, or younger than 65 with a qualifying disability. Over 65 million Americans are signed up for Medicare in the United States as of 2024, which displays the public’s dependence on this program to achieve their medical care [5]. CI procedures have been covered by Medicare in some form since 2005; however, out-of-pocket costs can heavily influence a person’s decision in completing these procedures [6]. 

Ambulatory Surgery Centers (ASCs) are an alternative that the public can use to access cheaper and efficient outpatient surgical care compared to Hospital Outpatient Departments (HOPDs). ASCs mostly provide uncomplicated surgical care that does not require an inpatient stay or long-term follow-up, which includes procedures like CI [7]. However, although ASCs on average provide procedures at a lower cost, health insurance reimbursement may be different depending on the medical home providing the surgery [8]. Via national averages provided by Medicare.gov, in 2024, the average cost for a CIS in an ASC was around $28,262, whereas HOPDs charged on average $31,851 [8]. However, with Medicare providing less reimbursement to those completing their care at ASCs, patients on average were expected to pay around $5,891 out of pocket to complete their procedures at an ASC as opposed to $1,871 in an outpatient hospital setting [8]. This highlights a historic difference in Medicare reimbursement between ASCs and HOPDs.

The Center of Medicare and Medicaid Services (CMS) specifically reimburses HOPDs via the Outpatient Prospective Payment System (OPPS), and ASCs via a similar ASC Fee Schedule system. CMS tends to favor higher reimbursements to HOPDs compared to ASCs, largely due to a decreased facility fee for an ASC, which holds fewer workers than a hospital [9]. Site-neutral payment has been a concept discussed in recent decades in order to achieve similar costs of medical procedures across different medical settings. Medicare has made some advancements in achieving site-neutral payments, particularly in 2019, by changing reimbursement rates of ASCs, which were previously subjected to change via the consumer price index (CPI), to match HOPD rates, which have historically been defined by the hospital market basket [10].

However, even with Medicare making this change, there seems to be a consistent difference in site-specific payments for certain procedures, which will likely influence a patient’s decision in their medical care. When it comes to CI procedures specifically, there have been few analyses on how the impact of the CMS’s updated ASC Fee Schedule has affected a physician’s reimbursement and a patient’s out-of-pocket costs. This study specifically aims to analyze the difference in reimbursement patterns for those covered through Medicare when receiving care from ASCs compared to HOPDs. It achieves this goal through a retrospective analysis of reimbursement patterns since CMS updated its ASC Fee Schedule from 2019 to 2021.

## Materials and methods

This retrospective cross-sectional study analyzed Medicare reimbursement patterns for cochlear implant surgery performed at ASCs and HOPDs. Medicare claims data spanning from January 1, 2019, to December 31, 2021, were utilized. The study focused on the reimbursement differences associated with CPT code 69930 for cochlear implant surgery performed in ASCs and APC code 5166, which is used in outpatient hospital settings. The study adhered to guidelines set forth by the Strengthening and Reporting of Observational Studies in Epidemiology (STROBE).

Data source and population

Medicare reimbursement data were obtained from the CMS database. The CMS database provides comprehensive information on past healthcare provider claims, including detailed payment structures listed by procedure type and healthcare setting. Claims that fell under Medicare Part B reimbursements and excluded any payments made by supplemental insurance or patient out-of-pocket costs were then assessed. The study population included patients who underwent cochlear implantation surgery from January 1, 2019, to December 31, 2021, as identified by CPT (69930) and APC (5166).

The inclusion criteria included patients 65 years or older enrolled in Medicare Part B during the study period, surgical procedures conducted either at ASCs or outpatient hospital settings, and availability of complete Medicare reimbursement data for cochlear implant surgery.

Exclusion criteria included procedures reimbursed under private insurance, patient-of-pocket costs, or Medicaid, ambiguous or incomplete CMS reimbursement records, or patients undergoing simultaneous procedures not directly related to cochlear implantation surgery.

Outcome measures

The primary outcome variable was the average Medicare reimbursement amount for cochlear implantation surgery, categorized by location. For ASCs, reimbursements were captured under CPT code 69930, and for outpatient hospital settings, reimbursements were captured under APC code 5166, which aggregates both facility fees and physician fees.

This variable represented the mean Medicare payment for each procedure, adjusted to 2021 dollars using the CPI for medical services. A secondary outcome is the annual trends in average reimbursement amounts for cochlear implantation surgery across both ASCs and outpatient hospital settings.

Statistical analysis

Unpaired two-tailed t-tests and box-and-whisker plots were used to compare average Medicare reimbursements between ASCs and outpatient hospital settings. The use of unpaired two-tailed t tests was appropriate due to the independent nature of the two provider types, along with the continuity of reimbursement data. Box-and-whisker plots served as a visual tool to complement the statistical analysis and provide a clear depiction of the variability of reimbursement across provider types.

The unpaired two-tailed t-tests were used to evaluate whether there were statistically significant differences in mean reimbursement amounts between the two settings for each year in the study period (2019, 2020, and 2021). A p-value < 0.05 was considered statistically significant. Separate unpaired two-tailed t-tests were done for each year to independently compare reimbursement means for ASCs and outpatient hospital settings. Box-and-whisker plots were used to illustrate the distribution of reimbursement amounts for the two provider types, and outliers were plotted individually to underline reimbursement variability.

## Results

Outpatient hospital settings

In 2019, outpatient hospital settings received a mean of 29595.58 dollars (SD = 3934.898). In 2020, they received a mean of 31464.87 (SD = 4177.054). Finally, in 2021, they received a mean of 32549.87 (SD = 4402.242).

ASC 

In 2019, ASC received a mean of 23202.01 dollars (SD = 3934.898). In 2020, they received a mean of 24986.88 (SD = 3367.238). Finally, in 2021, they received a mean of 26028.86 (SD = 3184.769).

**Figure 1:**
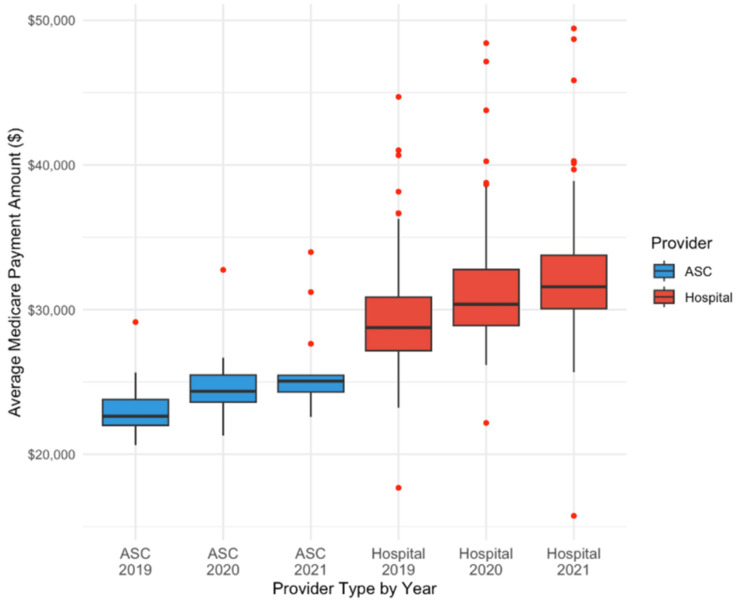
Medicare reimbursements: ASC vs hospital outpatient (2019-2021)

These results combined demonstrate that outpatient hospital settings received significantly greater reimbursements for cochlear implant surgery between 2019 and 2021 (p < 0.0001). For 2019, it was 28% higher, 26% higher in 2020, and 25% higher in 2021. Figure 1 further depicts these differences over time.

## Discussion

In this retrospective, cross-sectional analysis investigating discrepancies in Medicare reimbursement for cochlear implant surgery between 2019 and 2021 in outpatient settings versus ASCs, there was a significantly larger reimbursement amount in outpatient hospital settings compared to ASCs. There may be multiple reasons for this observed phenomenon.

Medicare accreditation

Previous literature from 2013 highlights a significant increase in the patient volume in outpatient surgery within the last three decades, nearly tripling in number [11]. As time progresses, procedures have shifted largely from being performed in an outpatient setting to ASCs [12]. There has been a decrease in growth rate in the number of ASCs between 2012 and 2018 [13]. When observing otolaryngology procedures, previous cross-sectional analyses determined there was a 1.6% increase in otolaryngology procedures performed at ASCs, whereas a 6% decrease in outpatient hospital settings between 2010 and 2017 [14]. This does not correlate with the amount of physician Medicare reimbursements as outlined previously and could be partly due to the Medicare accreditation process, structure, and guidelines required for reimbursement.

Accreditation is a thorough process that involves multiple steps in order to provide effective and quality care, which includes identifying and establishing a governance structure, credentials, privileges, peer review process, quality program, risk management activities, infection control, standards of care, and ancillary services [15]. Ancillary services correlate with licensure, which also correlates with the CMS, which is necessary to obtain Medicare reimbursement [15]. As ASCs are standalone facilities primarily owned by physicians, reimbursement is an arduous and expensive process. On the other hand, outpatient hospital settings are integrated into hospitals, which are already established healthcare facilities with previous Medicare accreditation. Therefore, ASCs perform more fee-for-service and utilize private healthcare insurance more for cochlear implants, which explains the higher Medicare reimbursement amount in outpatient hospital settings compared to ASCs.

Operational and facility fees

As mentioned previously, ASCs accept more private health care insurance as opposed to public insurance such as Medicare. The costs of cochlear implants between ASCs and outpatient hospital settings vary. In previous literature analyzing private versus public insurance and its access to cochlear implants revealed that Medicare frequently neglects covering surgeon costs [16]. Thus, Medicare fails to pay for the surgeon's services at ASCs. Furthermore, private insurance fails to completely fund hospital devices, including cochlear implants [16]. This explains why hospital-associated outpatient clinics opt to accept public insurance and, therefore, will receive a higher Medicare reimbursement as observed in our results. Additionally, outpatient hospital settings charge higher prices for surgical procedures when compared to ASCs [17]. Significantly higher Medicare reimbursements were seen in outpatient hospital settings compared to ASCs in our results.

Population and treatment complexity

As of 2024, nearly 66 million patients receive health insurance through Medicare [18]. As mentioned previously, Medicare primarily serves patients above the age of 65 or younger if they have a qualifying disability [5]. Old age is one of the strongest risk factors for hearing loss, affecting nearly two-thirds of adults over 60 [19,20]. Like hearing loss, with old age, the prevalence of comorbidities such as hypertension, hyperlipidemia, ischemic heart disease, and diabetes increases [21]. Therefore, hearing loss, in addition to multiple comorbidities, becomes more complex to treat and manage in a standalone ambulatory surgical center. Thus, being hospital-affiliated, outpatient hospital settings are more equipped to serve these complex patient populations and bill higher rates to manage these complexities to account for potential iatrogenic errors, which accounts for the higher Medicare reimbursements.

Cochlear implantation costs: ASCs versus hospital settings

The total costs charged to patients receiving cochlear implantation in an ASC versus an outpatient hospital setting vary drastically. According to the most recent procedure price lookup for outpatient services for cochlear implantation surgery with or without a mastoidectomy, patients pay an average of $1,871 if they undergo the procedure at an outpatient hospital setting versus $5,891 if performed at an ASC [22]. When using Medicare, if the cochlear implantation surgery is performed in a hospital-based setting or ASC, the surgery is covered under different parts of Medicare, further affecting the reimbursement amount [23]. Attributable to these observations, patients seek to receive cochlear implantation surgery at outpatient hospital settings as opposed to ASCs, increasing patient load and therefore higher Medicare reimbursements.

Geographical and socioeconomic impact on cochlear implants

Geographical and socioeconomic factors could have also impacted hearing impairment and the need for cochlear implants. In previous reviews discussing disparities associated with CIs, they highlighted how neighborhood and physical environment, which includes areas with exposure to markedly elevated noise exposure and poor living conditions, may influence and expedite the hearing loss, such as urban, inner cities [24], further impacting the necessity of CI. Additionally, this disparity affects specific geographical areas in which healthcare infrastructure, such as Medicare, directly impacts access to CIs [24].

In previous retrospective reviews analyzing factors affecting duration prior to CI, older age was noted to significantly prolong time to surgery (p = 0.038) [25]. The mean age in this study was 61 years, close to the age qualifying for Medicare. Additionally, there was a significantly longer delay to implantation in non-white patients compared to white patients with progressive hearing loss [25], further indicating socioeconomic barriers to CI.

Limitations 

The study only includes claims from Medicare; therefore, any procedures performed at non-accredited facilities were not included, which represents a limitation of our study. Additionally, there is an absence of patient-level or geographic adjustment due to the lack of this data being accessible on publicly available datasets, which could influence reimbursement variability. 

## Conclusions

This analysis demonstrates a clear and consistent quantitative disparity in Medicare reimbursement for cochlear implant surgery between ambulatory surgery centers (ASCs) and hospital outpatient departments (HOPDs) from 2019 to 2021. Across all three years examined, HOPDs received approximately $6,000-$7,000 more in Medicare reimbursement per procedure than ASCs, with differences remaining highly statistically significant (p < 0.001). Specifically, mean reimbursements for HOPDs increased from $29,595 in 2019 to $32,549 in 2021, whereas ASCs received only $23,202 in 2019 to $26,028 in 2021 during the same period. These findings quantitatively confirm that Medicare payments for cochlear implant surgery are systematically higher in hospital-affiliated outpatient settings. The implications of these differences extend beyond reimbursement mechanics. Because ASCs generally operate at lower total procedural costs, the observed reimbursement gap reduces their relative cost-effectiveness under Medicare’s current fee schedules. For patients, this translates into substantially higher out-of-pocket costs when receiving the same procedure in an ASC, despite ASCs being designed to offer lower-cost surgical care. This dynamic may disproportionately burden Medicare beneficiaries with lower socioeconomic status, thereby reinforcing existing inequities in access to cochlear implantation--an intervention already underutilized by populations with fewer financial and structural resources. Furthermore, unequal reimbursement may influence surgeon practice patterns and site-of-service selection, potentially shifting more Medicare patients toward hospital settings even when clinical complexity does not necessitate higher-cost care. This perpetuates system-level inefficiencies and may contribute to geographic disparities, as access to HOPDs is not uniformly distributed across regions. 

In summary, the quantitative findings of this study highlight a persistent and meaningful reimbursement imbalance that affects both cost-effectiveness at the system level and equity in care delivery for Medicare beneficiaries. Future research should evaluate whether these discrepancies reflect justified differences in patient complexity or represent modifiable policy artifacts within Medicare’s payment frameworks. Addressing these questions is essential for informing site-neutral payment reforms and ensuring equitable, cost-efficient access to cochlear implant surgery.
